# Surgery and outcomes of six patients with intradural epidermoid cysts in the lumbar spine

**DOI:** 10.1186/1477-7819-12-50

**Published:** 2014-03-04

**Authors:** Huabin Yin, Dan Zhang, Zhipeng Wu, Wang Zhou, Jianru Xiao

**Affiliations:** 1 Department of Bone Tumor Surgery, Changzheng Hospital, Second Military Medical University, Shanghai, China; 2 Department of Orthopedics, 149 Hospital, Lianyungang, Jiangsu, China

**Keywords:** retrospective study, epidermoid cyst, lumbar spine, case series, surgery, total excision

## Abstract

**Study design:**

This was a retrospective study.

**Objective:**

The aim of this study was to analyze the clinical characteristics and discuss the treatment options for epidermoid cysts in the lumbar spine.

**Summary of background data:**

Epidermoid cysts are rare benign neoplasms, which account for less than 1% of all intraspinal tumors. Due to their rarity, there are only a few case reports in the literature. Complete excision is the recommended treatment for an epidermoid cyst, but this is difficult to achieve in the spine. In spite of their benign nature, local recurrence is not uncommon for spinal epidermoid cysts after incomplete excision.

**Methods:**

Six patients with an epidermoid cyst in the lumbar spine underwent surgical treatment in our center between 2004 and 2011. A total excision using microsurgical techniques and reconstruction was successfully undertaken in all patients. Clinical data and detailed pathologic results were retrospectively analyzed. All cases were followed up for a median time of more than 4 years. The clinical data and surgical efficacy were analyzed to suggest treatment options for epidermoid cysts in the lumbar spine on the basis of a literature review and our own experience.

**Results:**

The mean age of the patients in this study was 37.7 years and the mean duration of pre-operative symptoms was 29.7 months (2 to 120 months). All patients were disease-free during their follow-up period. Radicular pain nearly disappeared, and patients suffering from neurologic deficits and defecation disorders recovered well.

**Conclusions:**

Although an epidermoid cyst is a benign tumor, it is apt to recur locally following inadequate removal. Therefore, complete excision with preservation of neural function is an ideal protocol for intraspinal epidermoid cysts. Microsurgical techniques are very useful.

## Background

Epidermoid cysts are rare benign neoplasms within the neuraxis, which are commonly located in the intracranial region and account for less than 1% of all intraspinal tumors [1,2]. In 1835, Cruveilhier was the first to describe epidermoid cysts, calling them *tumeurs perlées* (pearly tumors) [3,4]. Epidermoid cysts can be congenital or acquired. Congenital epidermoid cysts are frequently found in association with spinal dysraphisms such as syringomyelia, dermal sinus and spina bifida, while the most common etiology for an acquired cyst is repeated lumbar puncture [2,5].

Although epidermoid cysts can be extradural, intradural or extramedullary, or intramedullary in the spine, the tumors are often intradural and extramedullary in the lumbosacral region [6]. The symptoms of an epidermoid cyst are usually non-specific. Neurologic symptoms such as progressive paraparesis, motor-sensory complaints and sphincter troubles cause great distress [2,4,5,7,8]. Patients with an epidermoid cyst usually suffer for a long time with symptoms, for an average time of 6 years, due to their slow-growing nature [2,4].

Because it is an indolent benign tumor, an epidermoid cyst can be cured by complete excision. However, complete resection is difficult to achieve because its capsule adheres to the spinal cord or nerve roots, so subtotal resection is usually the more common surgical objective [9].

In this series, six cases with an epidermoid cyst in the lumbar spine underwent surgery in our center. This retrospective study was performed to analyze the clinical characteristics and suggest treatment options for epidermoid cysts in the lumbar spine.

### Patients and methods

A total of seven patients with epidermoid cysts in the lumbar spine who received an operation in our department were identified from 2004 to 2011. One patient had already been subjected three times to incomplete tumor resections without adjunct therapy at other institutions and was admitted to our center due to tumor recurrence. The remaining six patients were regarded as ‘intact’ cases since they had not received any surgical intervention or any other treatment prior to the surgical treatment at our institution. The six intact patients were selected to be analyzed as a series and their clinical data were recorded. The criteria for surgery were sustained pain and a progressive neurologic deficit in the region affected by the tumor. Clinical data, including symptoms, signs, radiographic features, surgical information and detailed pathologic results, were carefully reviewed. In this retrospective study, we look at the clinical characteristics and treatment options for intraspinal epidermoid cysts on the basis of a literature review and our own experience. The participants’ rights regarding informed consent were fully respected and our research was also approved by the hospital ethics committee.

## Case presentation

### Patient features

Our study includes six patients (one male and five females) with an epidermoid cyst in the lumbar spine. At diagnosis, the patients’ ages ranged from 15 to 56 years (mean: 37.7 years), and 4/6 (66.7%) of the patients were over 40 (Table 1). None of the usual associated spinal dysraphisms, such as syringomyelia, dermal sinus and spina bifida, were found in the six patients. The patients had no medical history of lumbar punctures or trauma.

**Table 1:** Clinical data for six cases with an epidermoid cyst in the lumbar spine

**Case**	**Age (years)/sex**	**Location**	**Presentation**	**Pre-operation Frankel score**	**Resection mode**	**Instrumentation**	**Post-operation Frankel score**	**Follow-up (months)**	**Local recurrence**	**Last status**
1	55/F	L1-2	3 months of pain and neurologic deficit	D	Total excision	PF	E	106	No	NED
2	16/F	L3	2 months of pain and neurologic deficit	D	Total excision	PF	E	47	No	NED
3	41/M	L1-3	2 years of pain and neurologic deficit, 1 month of difficult defecation	D	Total excision	PF	E	32	No	NED
4	43/F	L3-5	10 years of pain and neurologic deficit, 1 year of urinary and fecal incontinence	D	Total excision	PF	E	18	No	NED
5	15/F	L3-4	5 months of pain and neurologic deficit	D	Total excision	PF	E	80	No	NED
6	56/F	L3-4	2 years of pain and difficult defecation	E	Total excision	PF	E	65	No	NED
Mean	37.7							58		

F, female; M, male; NED, no evidence of disease; PF, posterior fixation.

### Symptoms and neurological findings

A dull and localized pain with a neurologic deficit was the most common complaint, and the mean duration of symptoms was 29.7 months, ranging from 2 to 120 months. Five patients had motor-sensory complaints and three cases suffered from defecation disorders (50%).

### Radiological findings

**Figure 1:**
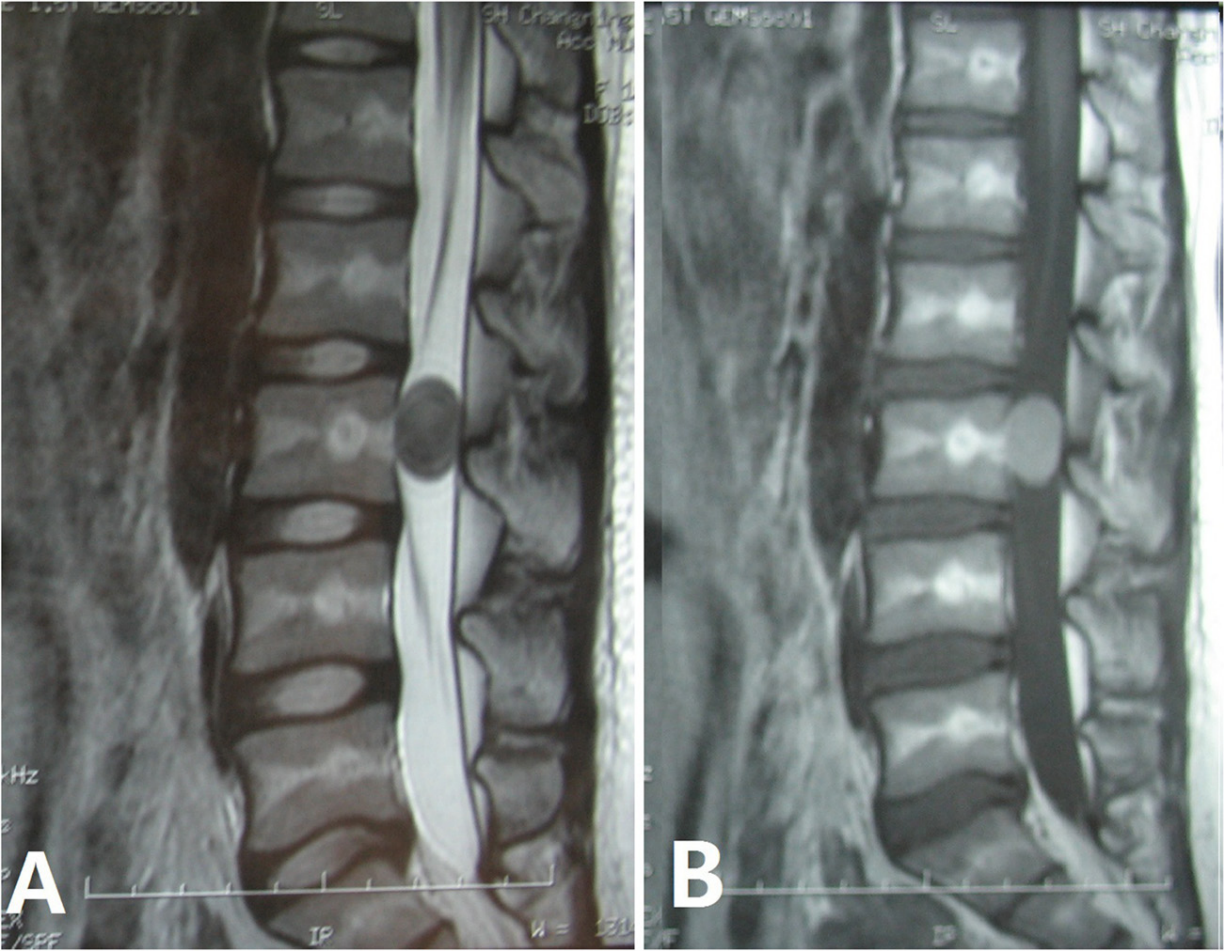
**MRI images of an epidermoid cyst with atypical imaging (case 2). (A)** T2-weighted image. The epidermoid cyst was hypointense. **(B)** T1-weighted image. The tumor was hyperintense.

The patients underwent X-rays, computerized tomography (CT) and magnetic resonance imaging (MRI) of the lumbar spine to give a radiological diagnosis. The plain radiographs were normal in all patients with an epidermoid cyst in the lumbar spine. CT showed a round or oval cyst containing liquid. On MRI, there were isointense (1 case) or hypointense (4 cases) regions in T1-weighted images and mixed hyperintense regions in T2-weighted images for five patients, while there were hyperintense regions in T1-weighted images and hypointense regions in T2-weighted images for one patient (case 2, Figure 1).

#### Staging findings

The cysts of the six patients were intradural and isolated lesions with no obvious satellite foci. There were no evidence of simultaneous similar intraspinal cysts in other segments. The numbers of patients where the tumor involved one segment, two segments and three segments were 1, 3 and 2, respectively.

#### Treatment and outcomes

For all patients, the tumors were excised using microsurgical techniques and reconstruction. Total excisions (the cyst was removed as a whole without capsule rupture) were successfully performed in all six cases. During surgery, the intraoperative blood loss ranged from 200 to 1400 mL (mean, approximately 533 mL). None of the patients died of surgical complications after surgery, and no recurrence was detected during the follow-ups. Moreover, there were no observed incidences of spine instability.

#### Pathology findings

**Figure 2:**
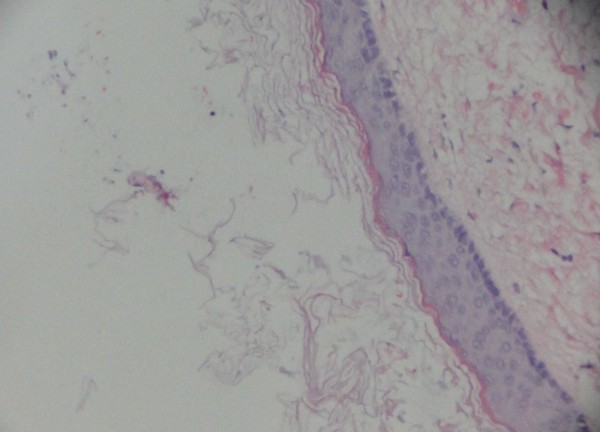
**Pathology image of an intraspinal epidermoid cyst.** The image shows well-differentiated stratified squamous epithelium lining the interior surface of the cyst capsule where keratin can be found.

Histologic diagnoses were obtained in all cases. Microscopic examination of the tumor specimens showed that the cyst walls were lined with compressed stratified squamous epithelium with abundant keratin material, which was consistent with epidermoid cysts (Figure 2).

#### Neurologic status

The pain experienced by all six patients was significantly reduced soon after surgery and was mostly absent by their 3-month follow-up visits. Their neurologic deficit and defecation disorders gradually improved. The patients’ Frankel scores decreased as well.

#### Follow-up evaluations

The mean follow-up period was 58 months (range: 18 to 106 months). The patients underwent X-rays, CT and MRI of the surgical segments involved, as well as adjacent vertebrae, after surgery. Follow-up data were also obtained from office visits and telephone interviews. In addition, neural function was re-evaluated 3 months after surgery according to the Frankel score system.

## Discussion

Epidermoid cysts in the spine are thought to arise from one of two possible origins: congenital or acquired [10]. Congenital epidermoid cysts, which are more common and often associated with other spinal dysraphisms, are caused by the anomalous implantation of ectodermal cells during closure of the neural tube between the third and fifth week of embryonic life [2,11,12]. The number of acquired cysts has decreased significantly in recent years. They are attributed to the displacement of epithelial tissue secondary to a previous lumbar puncture or trauma [13-15]. In our series, the epidermoid cysts in all six patients were regarded as congenital because they had neither suffered trauma nor received a lumbar puncture. Surprisingly, we also did not find evidence of other spinal abnormalities.

Due to the rarity of epidermoid cysts, there are no descriptions of the clinical features in the literature. In 1992, Roux *et al*. reviewed 47 cases with intramedullary epidermoid cysts of the spinal cord and summarized that: the average age was 34, the mean length of time between the onset of symptoms and diagnosis was 6 years and the ratio of males to females was 1.35:1 [2]. Our cohort has a similar age demographic (range: 15 to 56 years; mean: 37.7 years), much shorter duration of symptoms (range: 2 to 120 months; mean: 29.7 months) and a strange sex ratio (one man and five women). The shortened duration of symptoms is thought to be the result of significant progress in imaging technology, especially the wide application of MRI.

The symptoms and signs of an intraspinal epidermoid cyst are directly associated with the size and site of the tumor but are similar to those of other space-occupying lesions in the spinal column [4,16]. Non-specific symptoms and signs such as numbness, weakness, spasticity, paraparesis of lower extremities and defecation disorders pose challenges in pre-operative diagnosis. Ependymomas, metastasis, astrocytomas and dermoid cysts are alternative pre-operative diagnoses. With the help of MRI, ependymomas, metastasis and astrocytomas can be ruled out, but distinguishing an epidermoid cyst from a dermoid cyst relies on a pathological examination. For the six cases in our series, the long duration of localized pain and neurologic deficit as well as defecation disorders are non-specific and provide little help in the pre-operative diagnosis. Physical examinations and medical histories revealed no evidence of trauma or lumbar punctures.

MRI is an effective tool for the diagnosis of an intraspinal epidermoid cyst. X-rays often show a normal image and CT can only display the location and outline of the tumor. On MRI, the tumor is usually isointense or hypointense in T1-weighted images and hyperintense in T2-weighted images and the cyst content has the same signal as the cerebrospinal fluid [17-19]. But these signal characteristics are not always constant and some variations have been reported in the literature [18,20,21]. The MRI results for five patients in our series were consistent with the typical imaging findings reported in the literature while for one patient out of the six there were unusual images (Figure 1).

Pathologic examination is vital for the final diagnosis of an epidermoid cyst. Histologically, epidermoid cysts are composed of lined stratified squamous epithelium supported by an outer layer of collagenous tissue. Progressive desquamation of keratin from the epithelial lining toward the interior of the cyst produces a soft white material rich in cholesterol crystals [2,22-24]. The absence of skin adnexa is the key to differentiate one from a dermoid cyst [2,24].

A cyst contains considerable amounts of fat and little cholesterol, which may produce a vigorous inflammatory reaction if the cyst ruptures. Two cases reported in the literature illustrate this point [2,17,19,25].

We found an interesting phenomenon. All the cases of intraspinal epidermoid cysts in our series and cases in the literature were isolated and single, while the dermoid cysts in the spine with a similar pathogenesis and pathological manifestation often had satellite lesions at adjacent levels [2,9]. According to Willis, epidermoid cysts and dermoid cysts are two subtypes of epidermal inclusion cysts, which are neoplasms derived from sequestered skin [26]. Several theories have attempted to explain the pathogenesis of epidermal inclusion cysts. The classic, widely quoted theory set out by von Bostroem in 1897 expounded that those were developmental errors of the neural tube, occurring between the third and fifth week of embryonic life [12]. A newborn rat model developed by Van Gilder and Schwartz had skin homografts implanted along the neuraxis, which transformed themselves into epidermoid cysts or dermoid cysts. This work added weight to the theory of von Bostroem [27]. The theory also considers that the timing of the event (early or late) determines the type of tumor (dermoid or epidermoid) that will form [9,12]. We have reviewed other theories for the pathogenesis of both types of tumor and regretfully found that no theory had tried to explain the phenomenon we found. In our opinion, the timing of developmental errors and the involvement of skin adnexa may be related to the difference between these two similar types of tumor. Perhaps, multiple epidermoid cysts in the spine, which are rarer, have not yet been found and reported.

Given their indolent, benign nature, asymptomatic epidermoid cysts should be managed conservatively. Surgical excision is essential for lesions with symptomatic progression and where imaging shows that neural elements are compressed [9,22,24,28]. Although radiotherapy has been used in one case with repeated relapses of epidermoid cysts, achieving a relatively good result, this treatment should only be used for patients who refuse surgery or are inoperable for medical reasons [29]. Undoubtedly complete excision without neural damage is the goal of treatment. Emptying of the cyst material can be performed easily but the intimate adherence between the capsule and the spinal cord makes this goal difficult. So subtotal excision tends to be the more common surgical pattern for avoiding possible neural damage [10,18,19,22,23,30]. Subtotal excision also causes great distress for patients and doctors because debris from the tumor may cause an early relapse of symptoms and the spread of cyst contents can cause foreign body reactions and severe complications [2,17,19,23,28,29].

Total excision, which requires complete removal of the tumor without rupturing the capsule, is the perfect form of complete excision. It has higher technical requirements and comes with a higher possibility of neural damage. Microsurgical techniques can help to achieve total excision and subsequently decrease the chance of a relapse. In our series, tumors in all six cases were located in the lumbar spine where total excision is easier to achieve and neural damage is more likely to be avoided using microsurgical techniques. The encouraging results are that radicular pain nearly disappeared, patients suffering from a neurologic deficit and defecation disorders recovered well and no patients suffered a relapse during their follow-up periods.

In spite of the indolent, benign nature of epidermoid cysts, local recurrence is not uncommon after incomplete surgical excision [2,17,19,23,28,29]. Metastatic lesions of an epidermoid cyst have never been reported. Malignant transformations have not been found in the spine but there have been a few reports of intracranial cases with a malignant transformation [31-35]. Symptomatic relapsed cases should be retreated by surgical excision. Because of the formation of scar tissue resulting from previous interventions, complete excision is more difficult for relapsed cases and palliative approaches with the aim of relieving symptoms are often implemented.

## Conclusions

Although they are rare benign tumors, epidermoid cysts can occur intraspinally and cause severe neurological deficits with a long duration of symptoms. MRI is an effective tool for diagnosis with characteristic isointense or hypointense regions in T1-weighted images and hyperintense regions in T2-weighted images. However, pathologic examination is still the gold standard for final diagnosis. With the help of microsurgical techniques, total excision may lead to complete recovery for patients with symptomatic lesions. When total excision is impossible, subtotal excision can be adopted to avoid damaging neural function. Extreme care should be taken to minimize the residual and prevent the contents of the cyst spreading into the subarachnoid space, which can lead to prolonged chemical meningitis and subsequent arachnoiditis.

The pathogenesis of epidermoid cysts is still not very clear and the phenomenon that all intraspinal epidermoid cysts are isolated and single cannot be fully explained. More research is needed to clarify the pathogenesis of epidermoid cysts, which will also aid treatment.

## Consent

Written informed consent was obtained from each patient for publication of this study and the accompanying images.

## Abbreviations

CT: Computed tomography; MRI: Magnetic resonance imaging.

## Competing interests

The authors declare that they have no competing interests.

## Authors’ contributions

HY, WZ and JX conceived and designed the study. HY, DZ, ZW, WZ and JX collected the data. HY and JX wrote the paper. All authors read and approved the final manuscript.

## Authors’ information

HY, DZ and ZW are the co-first authors.

## References

[B1] Amato VG Assietti R Arienta C Intramedullary epidermoid cyst: preoperative diagnosis and surgical management after MRI introduction. Case report and updating of the literature J Neurosurg Sci 2002 46 122 126 12690335

[B2] Roux A Mercier C Larbrisseau A Dube LJ Dupuis C Del Carpio R Intramedullary epidermoid cysts of the spinal cord. Case report J Neurosurg 1992 76 3 528 533 10.3171/jns.1992.76.3.0528 1738035

[B3] Cruveilhier J Anatomie Pathologique 1835 2 Paris: Bailliere JB

[B4] Zavanone M Guerra P Rampini PM Crotti F Vaccari U A cervico-dorsal intramedullary epidermoid cyst. Case report and review of the literature J Neurosurg Sci 1991 35 2 111 115 1757803

[B5] Lunardi P Missori P Gagliardi FM Fortuna A Long-term results of the surgical treatment of spinal dermoid and epidermoid tumors Neurosurgery 1989 25 6 860 864 10.1227/00006123-198912000-00002 2601815

[B6] Bloomer CW Ackerman A Bhatia RG Imaging for spine tumors and new applications Top Magn Reson Imaging 2006 17 2 69 87 10.1097/RMR.0b013e31802bb38f 17198224

[B7] Jadhav RN Khan GM Palande DA Intramedullary epidermoid cyst in cervicodorsal spinal cord J Neurosurg 1999 90 1 Suppl 161 10413147 10.3171/spi.1999.90.1.0161

[B8] Tekkök IH Palaoglu S Erbengi A Onol B Intramedullary epidermoid cyst of the cervical spinal cord associated with an extraspinal neuroenteric cyst: case report Neurosurgery 1992 31 1 121 125 1641090 10.1097/00006123-199207000-00018

[B9] Ogden AT Khandji AG McCormick PC Kaiser MG Intramedullary inclusion cysts of the cervicothoracic junction. Report of two cases in adults and review of the literature J Neurosurg Spine 2007 7 2 236 242 10.3171/SPI-07/08/236 17688066

[B10] Scarrow AM Levy EI Gerszten PC Kulich SM Chu CT Welch WC Epidermoid cyst of the thoracic spine: case history Clin Neurol Neurosurg 2001 103 4 220 222 10.1016/S0303-8467(01)00156-1 11714565

[B11] Chandra PS Manjari T Devi BI Chandramouli BA Srikanth SG Shankar SK Intramedullary spinal epidermoid cyst Neurol India 2000 48 1 75 77 10751819

[B12] von Bostroem E Ueber die pialen Epidermoide. Dermoide und Lipome und duralen Dermoide Zentralbl Allg Pathol 1897 8 1 98

[B13] Baba H Wada M Tanaka Y Imura S Tomita K Intraspinal epidermoid after lumbar puncture Int Orthop 1994 18 2 116 118 10.1007/BF02484422 8039955

[B14] Gardner DJ O’Gorman AM Blundell JE Intraspinal epidermoid tumour: late complication of lumbar puncture CMAJ 1989 141 3 223 225 2752348 PMC1269411

[B15] Pear BL Iatrogenic intraspinal epidermoid sequestration cysts Radiology 1969 92 2 251 254 5765928 10.1148/92.2.251

[B16] Biliciler B Vatansever M Fuat Erten S Saraç K Colak A A huge intramedullary epidermoid cyst: mimicking cauda equina ependymoma. Diagnostic failure of myelography and myelo-CT J Neurosurg Sci 1996 40 2 149 152 9049900

[B17] Munshi A Talapatra K Ramadwar M Jalali R Spinal epidermoid cyst with sudden onset of paraplegia J Cancer Res Ther 2009 5 4 290 292 10.4103/0973-1482.59913 20160364

[B18] Teo BT Lin CC Chiou TL Chen SC Yen PS Unusual magnetic resonance characteristics of a cerebellopontine angle epidermoid cyst with upper cervical spinal canal extension J Clin Neurosci 2006 13 7 781 784 10.1016/j.jocn.2005.08.011 16723231

[B19] Yen CP Kung SS Kwan AL Howng SL Wang CJ Epidermoid cysts associated with thoracic meningocele Acta Neurochir (Wien) 2008 150 3 305 308 10.1007/s00701-007-1398-4 18193152

[B20] Gupta S Gupta RK Gujral RB Mittal P Kuriyal M Krishnani N Signal intensity patterns in intraspinal dermoids and epidermoids on MR imaging Clin Radiol 1993 48 6 405 413 10.1016/S0009-9260(05)81110-9 8293647

[B21] Visciani A Savoiardo M Balestrini MR Solero CL Iatrogenic intraspinal epidermoid tumor: myelo-CT and MRI diagnosis Neuroradiology 1989 31 3 273 275 10.1007/BF00344358 2779779

[B22] Fereydoonian NA Bakhti S Fereshtehnejad SM Tabibkhooei AR Intramedullary thoracic spine epidermoid cyst with myelopathic presentations: a report of a rare case Clin Neurol Neurosurg 2013 115 6 841 843 10.1016/j.clineuro.2012.08.002 22959213

[B23] Gonzalvo A Hall N McMahon JH Fabinyi GC Intramedullary spinal epidermoid cyst of the upper thoracic region J Clin Neurosci 2009 16 1 142 144 10.1016/j.jocn.2008.04.017 19013801

[B24] Kumar A Singh P Jain P Badole CM Intramedullary spinal epidermoid cyst of the cervicodorsal region: a rare entity J Pediatr Neurosci 2010 5 1 49 51 21042510 10.4103/1817-1745.66675 PMC2964782

[B25] Manno NJ Uihlein A Kernohan JW Intraspinal epidermoids J Neurosurg 1962 19 754 765 10.3171/jns.1962.19.9.0754 14469387

[B26] Willis RA The borderland of embryology and pathology Bull N Y Acad Med 1950 26 7 440 460 15426876 PMC1930017

[B27] Van Gilder JC Schwartz HG Growth of dermoids from skin implants to the nervous system and surrounding spaces of the newborn rat J Neurosurg 1967 26 1 14 20 5335180 10.3171/jns.1967.26.1part1.0014

[B28] Fleming C Kaliaperumal C O’Sullivan M Recurrent intramedullary epidermoid cyst of conus medullaris BMJ Case Rep 2011 doi:10.1136/bcr.11.2011.5090 10.1136/bcr.11.2011.5090 PMC3238133 22669964

[B29] Bretz A Van den Berge D Storme G Intraspinal epidermoid cyst successfully treated with radiotherapy: case report Neurosurgery 2003 53 6 1429 1431 14633311 10.1227/01.neu.0000093828.70768.40

[B30] Cataltepe O Berker M Akalan N A giant intramedullary spinal epidermoid cyst of the cervicothoracic region Pediatr Neurosurg 2004 40 3 120 123 10.1159/000079853 15367801

[B31] Chon KH Lee JM Koh EJ Choi HY Malignant transformation of an epidermoid cyst in the cerebellopontine angle J Korean Neurosurg Soc 2012 52 2 148 151 10.3340/jkns.2012.52.2.148 23091675 PMC3467374

[B32] Hamlat A Hua ZF Saikali S Laurent JF Gedouin D Ben-Hassel M Guegan Y Malignant transformation of intracranial epithelial cysts: systematic article review J Neurooncol 2005 74 2 187 194 10.1007/s11060-004-5175-4 16193391

[B33] Link MJ Cohen PL Breneman JC Tew JM Jr Malignant squamous degeneration of a cerebellopontine angle epidermoid tumor. Case report J Neurosurg 2002 97 5 1237 1243 10.3171/jns.2002.97.5.1237 12450053

[B34] Sawan B Vital A Loiseau H Dousset V Strub D Vital C Squamous cell carcinoma developing in an intracranial prepontine epidermoid cyst Ann Pathol 2000 20 3 258 260 10891726

[B35] Tamura K1 Aoyagi M Wakimoto H Tamaki M Yamamoto K Yamamoto M Ohno K Malignant transformation eight years after removal of a benign epidermoid cyst: a case report J Neurooncol 2006 79 1 67 72 10.1007/s11060-005-9117-6 16583265

